# Subclinical hyperthyroidism and dementia: the Sao Paulo Ageing & Health Study (SPAH)

**DOI:** 10.1186/1471-2458-10-298

**Published:** 2010-06-01

**Authors:** Isabela M Benseñor, Paulo A Lotufo, Paulo R Menezes, Márcia Scazufca

**Affiliations:** 1 Hospital Universitário, University of Sao Paulo, Sao Paulo, Brazil; 2 School of Medicine, University of Sao Paulo, Sao Paulo, Brazil; 3 Hospital das Clínicas, University of Sao Paulo, Sao Paulo, Brazil; 4 Hospital João Evangelista, São Paulo, Brazil

## Abstract

**Background:**

Several epidemiologic studies have shown a possible association between thyroid function and cognitive decline. Our aim was to evaluate the association of subclinical hyperthyroidism and dementia in a population sample of older people

**Methods:**

A cross-sectional study - São Paulo Ageing & Health Study (SPAH) - in a population sample of low-income elderly people ≥ 65 years-old to evaluate presence of subclinical thyroid disease as a risk factor for dementia. Thyroid function was assessed using thyrotropic hormone and free-thyroxine as well as routine use of thyroid hormones or antithyroid medications. Cases of dementia were assessed using a harmonized one-phase dementia diagnostic procedure by the "10/66 Dementia Research Group" including Alzheimer's disease and vascular dementia. Logistic regression models were used to test a possible association between subclinical hyperthyroidism and dementia.

**Results and discussion:**

Prevalence of dementia and of subclinical hyperthyroidism were respectively of 4.4% and 3.0%. After age adjustment, we found an association of subclinical hyperthyroidism and any type of dementia and vascular dementia (Odds Ratio, 4.1, 95% Confidence Interval [95% CI] 1.3-13.1, and 5.3 95% CI, 1.1-26.4; respectively). Analyzing data by gender, we found an association of subclinical hyperthyroidism with dementia and Alzheimer's disease only for men (OR, 8.0; 95% CI, 1.5-43.4; OR, 12.4; 95% CI, 1.2-128.4; respectively). No women with subclinical hypothyroidism presented Alzheimer's disease in the sample.

**Conclusion:**

The results suggest a consistent association among people with subclinical hyperthyroidism and dementia.

## Background

Thyroid disease and dementia are relatively common in elderly people. However, the diagnosis is difficult as symptoms can be vague and clinical presentation is confounded by the ageing process. Due to the close association between thyroid function and cognitive performance, the hypothesis thyroid dysfunction may be a risk factor for cognitive impairment has been investigated with conflicting results [1-10]. The Leiden-85 study did not show any association between levels of thyroid stimulating hormone (TSH) and free thyroxine (FT_4_) [1] with cognitive impairment. However, the Women's Health and Aging Study reported cognitive decline in women with low levels of total thyroxine (T_4_) over a three year period, although this trend was within the normal range for women [2].

In contrast to previous data, the Rotterdam Study determined that subclinical hyperthyroidism increased the risk of dementia and Alzheimer's disease three fold at follow-up in 1,893 participants [4], and the Kungsholmen Project identified an association between declining levels of TSH and a six year increase of memory deficit at follow-up [5]. The Rotterdam Scan Study did not show any association between TSH and thyroid hormone levels and the risk of dementia or Alzheimer's disease; however, nondemented subjects with higher FT_4 _levels exhibited greater hippocampal and amygdalar atrophy on magnetic resonance imaging [6]. The Honolulu-Asia Aging Study determined that higher total and FT_4 _levels were consistently associated with dementia, Alzheimer's disease, and neuropathology [7].

Recently, two prospective studies evaluated the relationship between thyroid function and risk of dementia and found an association in both directions. Hogevorst et al. found a positive association between hypothyroidism and cognitive impairment in a sample of elderly patients, as well as a positive association among participants with high-normal FT_4 _levels and cognitive impairment after two year follow-up [8]. The Framingham Study disclosed a positive association between women with serum thyrotropin in the lowest (< 1.0 mIU/L) or highest (> 2.1 mIU/L) tertiles of serum thyrotropin concentration and an increased risk of Alzheimer's disease, although no relationship was found between thyroid function and Alzheimer's disease in men [9]. Both studies suggest that low and high-normal TSH/FT_4 _levels can be associated with an increased risk of cognitive impairment or even Alzheimer's disease.

There have been few studies in Brazil concerning the prevalence of dementia, and no previous data has been gathered regarding the prevalence of thyroid dysfunction in population-based samples of older people. The present study is the first cross-sectional population-based study to explore the relationship of thyroid function and dementia, Alzheimer's disease, and vascular dementia in low-income, elderly people in the city of São Paulo, Brazil.

## Methods

A cross-sectional one-phase population-based study was carried out with all residents aged 65 years-old or older living in a economically deprived area of São Paulo, Brazil, to evaluate prevalence of dementia as part of a collaborative program developed by the 10/66 Dementia Research Group. Participants were those enrolled in the baseline assessment of the Sao Paulo Ageing & Health Study (SPAH) whose methodology can be assessed elsewhere [10,11]. The study was approved by the Institutional Review Board and all participants signed an informed consent.

### Sample

The baseline assessment of the SPAH was carried out with all people aged 65 years or older, residents in 66 pre-determined census sectors (catchment areas) in the neighborhood of Butantan located in the Western side of the city of São Paulo. Study participants lived in shantytowns or households covered by primary care units from the Family Health Program, with the lowest family income in the area. A total of 2,072 persons (91.4% of those invited) were recruited through systematic door knocking and were included in the SPAH. As the investigation of thyroid disorders started after the baseline assessment of the SPAH was underway, 699 [33.7%] participants included in the SPAH with no data about thyroid function were excluded from the present study. There was no difference regarding age, sex, socio-demographic, and anthropometric variables between the participants included in the thyroid function study as compared to the 699 non-participants. We have also excluded from the analysis 97 patients with clinical thyroid dysfunction or using medication for thyroid disorders, totaling 1,276 participants.

### Procedures

The study was conducted over a two-year period, from May 2003 to April 2005. All those aged at 65 years or over living in the study catchment area who accepted to participate in the study were interviewed. This meant that in households with two or more elderly people, all of them were invited to participate. All participants were assessed for socioeconomic characteristics.

Venous blood sample was obtained after an overnight fast. The serum obtained after centrifugation was used for hormone and biochemistry measurements. Thyroid stimulating hormone (TSH) concentration was measured by immunometric assay (kit AutoDelfiahTSH) and free-thyroxin (FT_4_) concentration was also determined using an immunometric assay (kit AutoDelfia T_4_). Anti-thyroid peroxidase (TpoAb) antibodies concentration was measured by radioimmunoassay, kit TpoAb RIA C.T. (BC 1018).

Thyroid dysfunction was assessed using TSH and F-T4 as well as routine use of thyroid hormones or antithyroid medications. Cut-off levels for TSH were < 0.4 μIU/ml for hyperthyroidism and > 4.0 μIU/ml for hypothyroidism. Cut-off levels for free-T4 were < 0.8 ng/dl for hypothyroidism and > 1.9 ng/dl for hyperthyroidism. According to both hormones, people in the sample were classified in five categories: clinical hyperthyroidism (low serum TSH combined with high levels of FT_4_), subclinical hyperthyroidism (low serum TSH with normal levels of F-T_4_), euthyroidism (normal TSH and normal FT_4_), subclinical hypothyroidism (high TSH with normal FT_4_) and clinical hypothyroidism (high TSH combined with low levels of FT_4_).

### Assessment of dementia

Dementia was assessed using a harmonized one-phase dementia diagnostic procedure by the 10/66 Dementia Research Group and validated for use in population-based studies in low and middle-income countries [11]. The diagnosis of dementia followed DSM-IV criteria [12]. The procedure includes assessment of cognitive function with the Community Screening Instrument for Dementia (CSI-D), [13] an adapted version of the *C*onsortium to *E*stablish a *R*egistry for *A*lzheimer's *D*isease (CERAD) ten word list learning task with delayed recall and animal verbal fluency, [14] a structured clinical mental state interview with the Geriatric Mental State (GMS) [15,16], a structured neurological assessment of localizing signs, parkinsonism, ataxia, apraxia and primitive "release" reflexes, assessment of participants' daily functioning and general health with the CSI-D, and of functional and cognitive decline with the History and Aetiology Schedule Dementia Diagnosis and Subtype (HAS-DDS) [17]. An algorithm combines data from all assessments and classifies participants as cases of dementia or not, and by dementia subtype (Alzheimer's disease, vascular dementia, mixed and others) [10,11,18].

Age was classified in four age strata: 65-69, 70-74, 75-79 and ≥ 80 years-old. Education was classified as: no formal education or one or more years of formal education. Monthly income was classified as < US$ 127 or ≥ US$ 127. Hypertension was defined if the participant had a medical history of hypertension, used medication for hypertension treatment or presented a systolic blood pressure > 140 mm Hg and/or diastolic blood pressure > 90 mm Hg [19] Diabetes was defined if the participant had a medical history of diabetes, used medication for diabetes treatment or presented a fasting blood glucose ≥ 126 mg/dl [20].

### Data analysis

Data entry was carried out twice using the program EPIDATA 3.0, and the validity check was carried out to identify and correct data entry errors. Data was analyzed using SPSS 15.0. Chi-square tests were used for comparison when appropriate. For continuous variables with normal distribution we used ANOVA. A logistic model was used to assess the association of subclinical hyperthyroidism with dementia, Alzheimer's disease and vascular dementia crude, adjusted by age, with a multivariate adjustment for age and BMI. TSH and free-thyroxine values were divided into five groups and a logistic model adjusted for age was performed using the third quintile as the reference. This was also performed for TSH and FT_4 _values within the normal range. Ninety-five percent confidence intervals were provided. A *P *value less than 0.05 was considered statistically significant.

## Results

**Figure 1:**
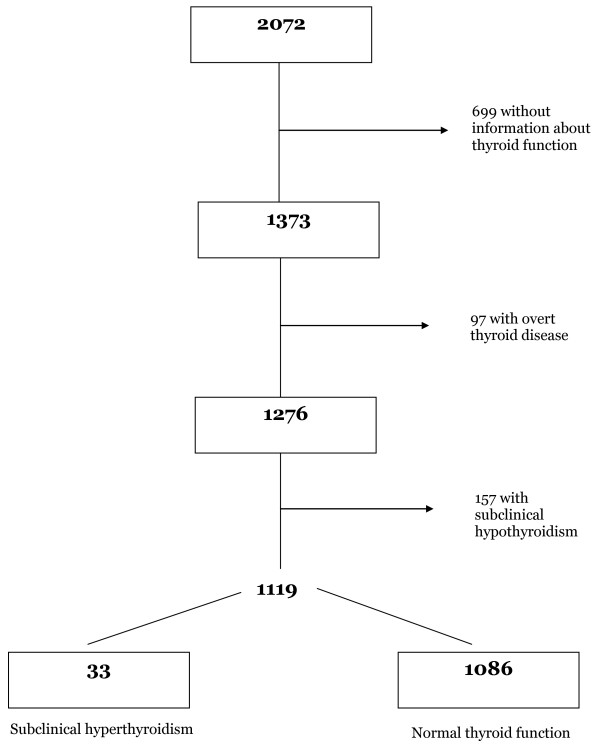
**Participants of the São Paulo Ageing & Health Study included in the analysis**.

Of the 1276 participants, 1086 presented normal thyroid function, 33 subclinical hyperthyroidism and 157 subclinical hypothyroidism (Figure 1). Mean (standard deviation) TSH values for elderly people with subclinical hyperthyroidism and subclinical hypothyroidism were 0.15 μIU/ml (0.12) with a range 0.0-0.3, and 6.6 μIU/ml (3.4) with a range of 4.1-25.3, respectively; mean FT_4 _(standard deviation) for elderly people with subclinical hyperthyroidism and subclinical hypothyroidism was 1.22 ng/dl (0.22) with a range of 0.89-1.67, and 0.96 ng/dl (0.15) with a range of 0.8-1.8, respectively. There were 49 cases of dementia, including 19 (38.8%) cases of Alzheimer's disease, 18 (36.7%) vascular, 9 (18.4%) mixed, and 3 (6.1%) cases of all other causes of dementia. Regarding the severity of dementia, 29 (59.2%) cases were mild, 12 (24.5%) moderate, and 8 (16.3%) cases were severe. Table 1 shows general characteristics of participants with and without dementia in elderly people with subclinical hyperthyroidism and normal thyroid function. Participants with dementia identified were older, leaner and with a higher rate of high blood pressure compared to people without dementia.

**Table 1:** General characteristics of participants according to presence or not of dementia.

	Presence of dementia		
		
Characteristics	Yes (N = 49)	No (N = 1070)	*P*
Age (years)*	78.5 (8.0)	71.9 (6.1)	< 0.001
Proportion of women (%)	33 (67.3)	644 (60.2)	0.20
Race			
White	13 (56.5)	530 (52.4)	0.81
Mixed	6 (26.1)	328 (32.4)	
Black	4 (17.4)	154 (15.2)	
Education (years)			
No formal education	24 (50.0)	422 (39.6)	0.10
≥ 1 year of formal education	24 (50.0)	645 (60.4)	
Monthly income			
< 127 US$	39 (79.6)	567 (53.0)	< 0.001
≥ 127 US$	10 (20.4)	503 (47.0)	
BMI (kg/m^2^)*	22.1 (4.3)	25.8 (5.0)	< 0.001
TSH (mU/l)*	1.4 (0.9)	1.7 (0.9)	0.04
Free-T4 (ng/dl)*	1.2 (1.2)	1.1 (0.4)	0.052
TPOAb positive antibodies (UI/ml)*	17.3 (12.9)	26.1 (81.5)	0.63
high blood pressure (%) †	42 (91.3)	822 (78.4)	0.02
diabetes (%)‡	12 (26.1)	21.9 (224)	0.31
Total cholesterol (mg/dl)	203 (42)	210 (44)	0.29
HDL-cholesterol (mg/dl)	59 (18)	56 (15)	0.13
LDL-cholesterol (mg/dl)	116 (32)	125 (35)	0.09
Current smokers (%)	6 (23.1)	153 (24.6)	0.54
History of alcohol abuse (%)	11 (22.4)	225 (21.0)	0.46

*Mean (standard deviation).

†Diagnosis of high blood pressure was done if positive medical history of high blood pressure or treatment for high blood pressure or systolic blood pressure > 140 mm Hg or diastolic blood pressure > 90 mm Hg.

‡Diagnosis of diabetes if positive medical history of diabetes or treatment for diabetes or fasting blood glucose > 126 mg/dl.

Table 2 shows the odds ratio of presence of any type of dementia, Alzheimer's disease or vascular dementia among people with subclinical hyperthyroidism using as reference people with normal thyroid function. After adjustment for age, we found an odds ratio (and 95% confidence interval) of subclinical hyperthyroidism with any type of dementia of 4.1 (95% CI, 1.3-13.1). We also found a positive association between subclinical hyperthyroidism and vascular dementia with an OR of 5.3 (1.1-26.4). Both results persisted significant after age and BMI adjustment. No association was found between subclinical hyperthyroidism and Alzheimer's disease. Analyzing data according to gender, after age-adjustment there was a consistent positive relationship between subclinical hyperthyroidism with dementia (OR, 8.0; 95% CI, 1.5-43.4) and with Alzheimer's disease (OR, 12.4; 95% CI, 1.2-128.4) in men. Both results persisted significant after age and BMI adjustment. No women with subclinical hyperthyroidism present Alzheimer's disease in the sample. No association was found between women with subclinical hyperthyroidism with any type of dementia or vascular dementia in the sample.

**Table 2:** Age-adjusted OR(95%CI) of dementia and its subtypes in people with subclinical hyperthyroidism compared to normal thyroid function.

	Model	Odds ratio (95% CI)
All (n = 33) subclinical hyperthyroidism		

Any type of dementia (n = 49)	Crude	3.2 (1.1-9.5)
	Age-adjusted	4.1 (1.3-13.1)
	Multivariate adjusted*	4.9 (1.5-15.7)
Alzheimer Disease (n = 19)	Crude	1.9 (0.2-14.3)
	Age-adjusted	2.1 (0.3-17.3)
	Multivariate adjusted*	2.5 (0.3-20.8)
Vascular dementia (n = 18)	Crude	4.3 (1.0-19.6)
	Age-adjusted	5.3 (1.1-26.4)
	Multivariate adjusted*	6.7 (1.4-33.1)

Men (n = 10) subclinical hyperthyroidism		

Any type of dementia (n = 16)	Crude	7.5 (1.5-38.4)
	Age-adjusted	8.0 (1.5-43.4)
	Multivariate adjusted*	8.1 (1.5-44.6)
Alzheimer Disease (n = 5)	Crude	11.9 (1.2-117.0)
	Age-adjusted	12.4 (1.2-128.4)
	Multivariate adjusted*	11.6 (1.1-120.7)
Vascular dementia (n = 9)	Crude	5.9 (0.7-52.0)
	Age-adjusted	6.1 (0.7-56.3)
	Multivariate adjusted*	5.8 (0.6-54.4)

Women (n = 23) subclinical hyperthyroidism)		

Any type of dementia (n = 33)	Crude	1.9 (0.4-8.5)
	Age-adjusted	2.6 (0.5-13.6)
	Multivariate adjusted*	3.4 (0.7-17.3)
Vascular dementia (n = 9)	Crude	3.7 (0.4-30.6)
	Age-adjusted	5.0 (0.5-55.0)
	Multivariate adjusted*	9.2 (0.9-96.4)

*Multivariate adjusted include adjustment for age and body-mass index.

We also performed the same analysis on subjects with subclinical hypothyroidism to determine whether there was an association with dementia. However, in contrast to the analysis for subclinical hyperthyroidism, we did not find any positive results in our analysis of dementia with subclinical hypothyroidism (data not showed).

We have also divided all TSH and FT_4 _values into five groups. Using the third quintile as the reference, we observed that subjects in the lowest quintile of TSH presented an age-adjusted OR for dementia of 3.6 (95% CI, 1.4-8.9) and an OR for vascular dementia of 9.3 (95% CI, 1.1-75.5). Restricting the analysis to only TSH values within the normal range, and still using the third quintile as the reference, we did not observe any significant association between TSH levels in quintiles and dementia. The same analysis was performed for FT_4_, but we did not observe any significant association, even when the analysis was restricted to values within the normal range.

## Discussion

In this cross-sectional study, we found a consistent association among older adults with subclinical hyperthyroidism and any type of dementia and vascular dementia but not with Alzheimer's disease. Analyzing presence of dementia according to gender, after age and BMI adjustment, subclinical hyperthyroidism was positively associated with any type of dementia and Alzheimer's disease in men but not in women.

Most people with dementia live in low and middle income countries but there is very sparse research data about risk factors for dementia in these populations [18]. Although a study conducted in Nigeria showed a lower prevalence of dementia than in developed countries [21,22], studies carried out in Brazil [23,24], a middle income country, have found the prevalence of dementia to be similar to that found in Europe: 7.1% in an elderly community-dwelling Brazilian population [23,24] and 5.1% in a community sample of elderly low-income people using the 10/66 protocol [10,25].

One possible cause for dementia is iodine deficiency [18,26]. In recent years, some studies described the levels of serum TSH and FT4 in areas with borderline iodine intake as a U-shaped curve with an increase in risk from both low and high iodine intakes described in studies performed in The Netherlands [27], Denmark [28], and China [29]. Endemic goiter has been described in Brazil since the 16th Century [30-33]. Since the early 1940's, salt supplementation with iodine has been mandatory in endemic areas, and only since 1997 has Brazil been considered a country with adequate iodine supplementation. Approximately 80.3% and 85.3% of women and men, respectively, in this sample of subjects grew up in areas of low iodine intake, and they have likely had adequate or more than adequate iodine intake since 1996 [34-36]. This could explain the slightly higher frequency of subclinical thyroid disease in this sample (3.0% in this sample vs. 2.5% in a pooled-prevalence sample from several studies) [37], and presents a good opportunity to investigate the relationship between dementia and subclinical thyroid function.

Our cross-sectional data showed an association of subclinical hyperthyroidism with presence of dementia in agreement with previous prospective studies [5-7,9]. However, in our analysis we found a positive relationship of subclinical hyperthyroidism and any kind of dementia and vascular dementia, in contrast to data from the Rotterdam Study [5] in which a positive association was found for dementia and Alzheimer's disease; and from the Framingham Study [9] in which a positive association was found only for women in the lowest (< 1.0 mIU/l) and in the highest tertile (> 2.1 mIU/l) of thyrothropin compared to women in the middle tertile (respectively, RR, 2.39; 95% CI, 1.47-3.87 and RR 2.15; 95% CI, 1.31-3.52). Further analysis of our data according to gender showed a positive relationship among subclinical hyperthyroidism and Alzheimer's disease only for men. We did not analyze the association of subclinical hyperthyroidism and Alzheimer's disease because no women in the sample with a diagnosis of subclinical hyperthyroidism had Alzheimer's disease. However, our results are also different from the Framingham Heart Study in which no association was found between subclinical hyperthyroidism and Alzheimer's disease in men [9].

Some specific characteristics of the population included in the SPAH can help explain the high frequency of vascular dementia. Cardiovascular disease is the first cause of death in Brazil and the burden of stroke is very high in the country, particularly in people with low socioeconomic status [38,39]. The number of cases of Alzheimer's disease and vascular dementia in the present study were similar, whereas in most studies carried out in developed countries the prevalence of Alzheimer's disease is higher than the prevalence of other causes of dementia [7,9,39]. However, recent research data have also suggest that cardiovascular disease and Alzheimer's disease overlap frequently [40-42].

We did not observe any evidence of a U-shaped relationship of TSH and FT_4 _values with dementia or Alzheimer's disease. Restricting the analysis to TSH and FT_4 _values within the normal range did not change the results. After dividing TSH in groups, we only observe a positive association with dementia for subjects in the lowest quintile of TSH levels still using the third quintile as the reference. These results differed from Tan et al that found a U-shaped relationship between TSH levels within the normal range and Alzheimer's disease in women [9]; and from Hogervorst et al that found an association of subjects with high normal FT4 levels and an accelerated cognitive decline and probably dementia at follow-up [8].

Our study has some weakness and strengths. It is a cross-sectional study that can not bring information about causality. We do not have precise information about iodine intake along the life course, and the sample size is restricted for some analyses according to dementia subtypes. However, it is one of the first studies addressing thyroid function and dementia in a very low-income population using a standardized protocol. Dementia diagnosis was done according to 10/66 protocol using adequate instruments for the diagnosis of dementia in low and middle income countries [16,18]. This implies some degree of misclassification. However, the validation of this method, which included a subsample from our center, showed acceptable performance for its use in epidemiological investigations. We did not analyze the possible association of alterations in thyroid function and other subtypes of dementia aside from Alzheimer's and vascular dementia as the number of cases was too small, as would be expected in a population-based epidemiological study. Prevalence of both subclinical hyper- and hypothyroidism was also low. Thus, we cannot exclude chance bias in the results. The number of subjects is also low for an analysis according to dementia severity. Other strength of the study was the exclusion of participants using medicines for treatment of thyroid disorders.

## Conclusion

In this low-income sample of elderly people, we found a positive association of subclinical hyperthyroidism with dementia, and specifically with vascular dementia. In men, a positive association was found for dementia and Alzheimer's disease. One possible explanation is the high cardiovascular mortality in Brazil that could be associated with vascular dementia and possibly to Alzheimer's disease.

## Competing interests

Dr Bensenor, Dr Lotufo and Dr Menezes are recipients of a fellowship of Brazilian Research Council (Conselho Nacional de Pesquisa - CNPq, Brasília, Brazil)

## Authors' contributions

IJMB participated in the design, acquisition of data, analysis, and interpretation of results, writing and revision of the manuscript. PAL participated in the design, analysis, and interpretation of results, writing and revision of the manuscript. PRM participated in the design and revision of the manuscript. MS participated in the design, interpretation of results, writing and revision of the manuscript. All authors read and approved the final version of the manuscript.

## Pre-publication history

The pre-publication history for this paper can be accessed here:

http://www.biomedcentral.com/1471-2458/10/298/prepub
